# Diagnostic and follow-up performance of serological tests for different forms/courses of alveolar echinococcosis

**DOI:** 10.1016/j.fawpar.2019.e00055

**Published:** 2019-05-08

**Authors:** Bruno Gottstein, Anja Lachenmayer, Guido Beldi, Junhua Wang, Bernadette Merkle, Xuan Lan Vu, Ursula Kurath, Norbert Müller

**Affiliations:** a Institute of Parasitology, Department of Infectious Diseases and Pathobiology, Vetsuisse Faculty, University of Bern, Bern, Switzerland; b Institute for Infectious Diseases, Medical Faculty, University of Bern, Bern, Switzerland; c Department of Visceral Surgery and Medicine, Visceral Surgery, Inselspital University Hospital Bern and University Bern, Bern, Switzerland

**Keywords:** AE, Alveolar echinococcosis, CE, Cystic echinococcosis, ELISA, Enzyme-linked immunosorbent assay, Em, *Echinococcus multilocularis*, EmVF, *Echinococcus multilocularis* vesicular fluid, EgHF, *Echinococcus granulosus* hydatid fluid, FDG-PET/CT, fluorodeoxyglucose Positron Emission Tomography/Computed Tomography, MRI, Magnetic Resonance Imaging, US, Ultrasonography, ABZ, Albendazole, *Echinococcus multilocularis*, Follow-up serology, ELISA, Immunoblotting, Diagnosis

## Abstract

Diagnosis of alveolar echinococcosis (AE) is predominantly based on imaging procedures combined with immunodiagnostic testing. In the present study, we retrospectively analyzed the performance of four serological tests (EgHF-ELISA, Em2-ELISA, recEm18-ELISA and Em-Immunoblotting) for initial diagnosis and subsequent monitoring of AE patients. Overall, 101 AE patients were included, grouped according to treatment options and immune status as follows: (A) curative surgical treatment (n = 45 patients), (B) non-radical or palliative surgical treatment (n = 11), (C) benzimidazoles only (n = 20), (D) immunocompromised with radical surgical treatment (n = 11), (E) immunocompromised with benzimidazoles only (n = 4), and finally a group of 10 AE patients (F) that were considered to present so-called “abortive” lesions. Initial (i.e. pretreatment) ELISA-based diagnosis for patients in groups A to E revealed overall diagnostic sensitivities of 95% for EgHF, 86% for Em2, and 80% for recEm18, respectively. Comparatively, the diagnostic sensitivity of Em-Immunoblotting was higher with an overall value of 98%. In group F, only Em-Immunoblotting had an excellent diagnostic sensitivity (100%), whereas the ELISAs had poor sensitivities of 30% (EgHF- and Em2-ELISA) or even 0% (recEm18-ELISA).

Serological monitoring of AE patients showed a clear association between a curative development of disease (induced either by surgery or benzimidazole medication) and a negativization in the ELISAs. This effect was most pronounced for the recEm18-ELISA, where 56% negativized following diagnosis/treatment, as compared to 36% for the EgHF-ELISA, and 37% for the Em2-ELISA, respectively. After radical surgery, the mean time until negativization in the recEm18-ELISA was 2.4 years (SD 1.6). This was significantly shorter than the mean 3.9 years (SD 2.5) in those AE patients with non-radical, palliative surgery or ABZ treatment who were able to negativize during the study period (*p* = 0.048).

Conclusively, Em-Immunoblotting appears as the most sensitive test to diagnose active as well as inactive (“abortive”) AE-cases. The inclusion of the ELISAs completes the initial diagnostic picture and offers valuable additional information. Conversely, recEm18-ELISA appears as the currently best serological tool to monitor a regressive and putatively curative course of AE in treated patients.

## Highlights

• EgHF-ELISA plus Em-Immunoblotting results in most sensitive initial AE serodiagnosis.
• Em2- and recEm18-ELISA allow differentiation between AE and CE.
• recEm18-ELISA is currently the best monitoring test for assessing curative course of AE.
• Presumably inactive (“abortive”) AE cases were all positive by Em-Immunoblotting.

## 1 Introduction

Alveolar echinococcosis (AE) is a severe parasitic disease caused by the larval form, the metacestode, of *Echinococcus multilocularis*. Metacestodes develop primarily in the liver and often behave like malignant tumors (Eckert et al., 1983). Symptomatic AE is conventionally diagnosed by imaging procedures demonstrating the presence of various forms of the *E*. *multilocularis* metacestode-induced lesions in the liver tissue (Brunetti et al., 2010; Grüner et al., 2017; Wen et al., 2019). Confirmation of this initial diagnosis is routinely performed by serology. Very reliable assay systems are available, some of them commercially, for the sensitive and specific detection of *E*. *multilocularis*-specific antibodies (Grüner et al., 2017; Siles-Lucas et al., 2017; Wen et al., 2019). A widely accepted serodiagnostic strategy includes a primary screening method and one or more subsequent confirmatory test procedures (Reiter-Owona et al., 2009; Yamano et al., 2009; Grüner et al., 2017). Our laboratory uses an EgHF-ELISA (*E*. *granulosus* genotype G1 Hydatid Fluid) for primary immunodiagnosis of both clinical AE and CE (cystic echinococcosis). This ELISA offers a high diagnostic sensitivity: 95% for AE, and 91% for CE, respectively (Müller et al., 2007). For subsequent specification of this anti-*Echinococcus* antibody response, *E*. *multilocularis*-specific ELISAs using the Em2-antigen (94% specificity: Gottstein et al., 1983; Müller et al., 2007) and the recEm18-antigen (94% specificity: Ito et al., 2002; Xiao et al., 2003; Li et al., 2010) can be employed. With this protocol, a differential diagnosis between AE and CE can be achieved in 90–95% of the echinococcosis cases. Additionally, Em-Immunoblotting based on in vitro-produced EmVF (Vesicular Fluid) antigen contributes significantly to the diagnostic sensitivity (Müller et al., 2007). In combination with the Em2- and recEm18-ELISA, this immunoblot method allowed diagnosis of both clinical and subclinical (including abortive) cases of AE with a maximal sensitivity of nearly 100%. As most laboratories laos have to include CE in their immunodiagnostic spectrum, the initial diagnostic screening can be preferentially based upon inclusion of the EgHF-ELISA.

For the treatment of AE, there are principally two major options: either a radical surgical intervention, i.e. complete removal of parasitic lesions (Hillenbrand et al., 2017), or a long-term continuous therapy with benzimidazole medication (albendazole (ABZ) or mebendazole) (Stojkovic et al., 2014; Vuitton et al., 2016; Gottstein and Beldi, 2016). As medication acts rather parasitostatic than parasiticidal, drug administration usually is life-long in all those cases that could not be radically treated by surgery. A long-term follow-up is recommended for all AE patients, as relapses can occur even 20 years after benzimidazole medication (Ammann et al., 2004; Ammann et al., 2015; Hillenbrand et al., 2017). Monitoring involves a combination of imaging and serology. Regression of AE from an active to a stable or even cured stage was found to be associated with a progressive reduction of anti-Em18 serum antibodies (Ishikawa et al., 2009; Tappe et al., 2010; Bi et al., 2018). Extensive evaluations of the recEm18-ELISA revealed that serum anti-Em18 antibody levels rapidly decline to undetectable concentrations upon complete surgical removal of the parasite tissue (Ammann et al., 2004; Ishikawa et al., 2009; Tappe et al., 2009, Tappe et al., 2010; Bresson-Hadni et al., 2011; Bi et al., 2018). Thus, imaging in combination with recEm18-ELISA proved to be optimal to monitor AE patients. However, development and validation of new monitoring procedures, such as undertaken by Kaltenbach et al. (2015), may further advance this important area.

Spontaneous cure of AE leading to calcification of the parasitic lesions is possible, and it has been claimed that this phenomenon occurs more frequently than previously assumed (Bresson-Hadni et al., 1994; Vuitton et al., 2006). Such calcified lesions were shown to consist of remaining parasitic laminated layer, but do not anymore contain any living parasite tissue (Rausch et al., 1987). Cure has also been reported in some AE patients continuously treated with ABZ (Ammann et al., 2004; Crouzet et al., 2010). Immunosuppression on the other hand has been shown to allow for uncontrolled and metastasizing growth of the parasite (Sailer et al., 1997; Bresson-Hadni et al., 1999; Koch et al., 2003; Zingg et al., 2004). Furthermore, we do not yet know if the antibody response might be quantitatively or qualitatively altered in immunosuppressed patients, thus posing a challenge to serodiagnosis.

The present work is a retrospective analysis of the performance of in-house laboratory tests routinely used (a) to diagnose, and (b) to monitor different groups of AE patients, most of them having been hospitalized at the University Hospital in Bern, Switzerland: (A) AE patients with curative surgery treatment; (B) AE patients with non-radical or palliative surgical treatment; (C) AE patients with benzimidazole treatment only; (D) AE patients who had an immunosuppressive history or persisting condition prior to AE diagnosis, with radical surgical treatment; (E) AE patients who had an immunosuppressive history or persisting condition prior to AE diagnosis, some with benzimidazole treatment only, some with additional palliative interventions; (F) individual AE patients (non-diseased) who presented so-called “abortive” or “died-out” lesions.

## 2 Patients, materials and methods

### 2.1 AE patients

Selected laboratory and clinical data were retrospectively assessed from a total of 101 patients with AE. All initially presented and were subsequently treated and monitored at the University Hospital in Bern, except for 7 AE patients who were hospitalized and treated in regional hospitals. With their first hospitalization, all patients provided an informed consent for their serum samples to be diagnostically tested and subsequently stored at −80 °C for subsequent serological investigations. Each diagnosis was based on the WHO-IWGE criteria (Brunetti et al., 2010), which included staging according to the parasitic lesions/invasion of neighboring organs/metastasis (PNM) classification of AE lesions (Wang et al., 2011). All samples used in this study were anonymized.

All 101 AE patients were followed prospectively at minimum every 6 months for the first two years, and subsequently every year. Each follow-up evaluation included routine blood and liver function tests, measurements of blood levels of ABZ sulfoxide, as well as serological tests as described below. Ultrasonography (US) and computer-assisted tomography (CT) scanning was performed upon request of clinicians, as well as, in a restrictive manner, FDG-PET/CT scan. Magnetic resonance imaging (MRI) was performed only when specifically indicated. Suspected new residual lesions or increases in the size of existing recurrences/metastases were revealed by US and/or CT. Basically, AE patients were clinically managed and followed in the best possible way as consensus recommended (Brunetti et al., 2010; Kern, 2010; Wen et al., 2019).

The inclusion criteria for the present study were as follows: patients must present any kind of parasitic lesion compatible with *E*. *multilocularis*, including also calcified foci. Lesions, if clinically indicated, were confirmed either by core needle biopsy, diagnostic laparoscopy or surgical resection material that underwent histology and/or gDNA-based PCR (Trachsel et al., 2007).

The patients were grouped, according to their clinical status at diagnosis, as follows (see also Table 1):

(A) 45 AE patients with curative surgical treatment. One case included an orthotropic liver transplantation. Age of the patients at diagnosis ranged from 22 to 88 years (mean 42 years). The range of the follow-up time after diagnosis was from 1 to 15 years (mean 5 years).
(B) 11 AE patients with non-radical or palliative surgical treatment. From these, 8 patients had the liver affected only, one presented additional retroperitoneal lesions, one presented additional lung lesions, one additional lesions in the lungs, the diaphragm and the brain, and one additional lesions in the lungs, the pancreas and retroperitoneally. Age of the patients at diagnosis ranged from 24 to 75 years (mean 58 years). The range of the follow-up time after diagnosis was from 1 to 20 years (mean 6 years).
(C) 20 AE patients with benzimidazole only treatment. From these, 18 patients had the liver affected only, one presented additional retroperitoneal lesions, and one presented additional lung and brain lesions. Age of the patients at diagnosis ranged from 19 to 80 years (mean 53 years). The range of the follow-up time after diagnosis was from 2 to 23 years (mean 6 years).
(D) 11 immunosuppressed AE patients with radical surgical treatment. In all of these AE patients, the immunocompromised status was documented and thus occurred prior to AE diagnosis; details on the kind of immunosuppression/modulation are shown in Table 1. All 11 patients presented only hepatic lesions. Age of the patients at diagnosis ranged from 22 to 75 years (mean 58 years). The range of the follow-up time after diagnosis was from 2 to 7 years (mean 4 years).
(E) 4 immunosuppressed AE patients with benzimidazole only treatment. In all of these AE patients, the immunocompromised status was documented and thus occurred prior to AE diagnosis; details on the kind of immunosuppression/modulation are shown in Table 1. All 4 patients presented only one or a few intrahepatic lesions. Age of the patients at diagnosis ranged from 41 to 74 years (mean 68 years). The range of the follow-up time after diagnosis was from 3 to 8 years (mean 6 years).
(F) 10 AE patients (without clinical manifestations) that were considered to present so-called “abortive” lesions. In 3 out of these 10 patients, a surgical resection of the lesion was carried out, and the abortive status confirmed by histology. In another 2 out of these 10 patients, a core needle biopsy was taken for diagnostic purpose; AE was then confirmed by PCR (Trachsel et al., 2007). Age of the patients at diagnosis ranged from 42 to 88 years (mean 54 years). The range of the follow-up time after diagnosis was from 1 to 15 years (mean 5 years).

**Table 1:**
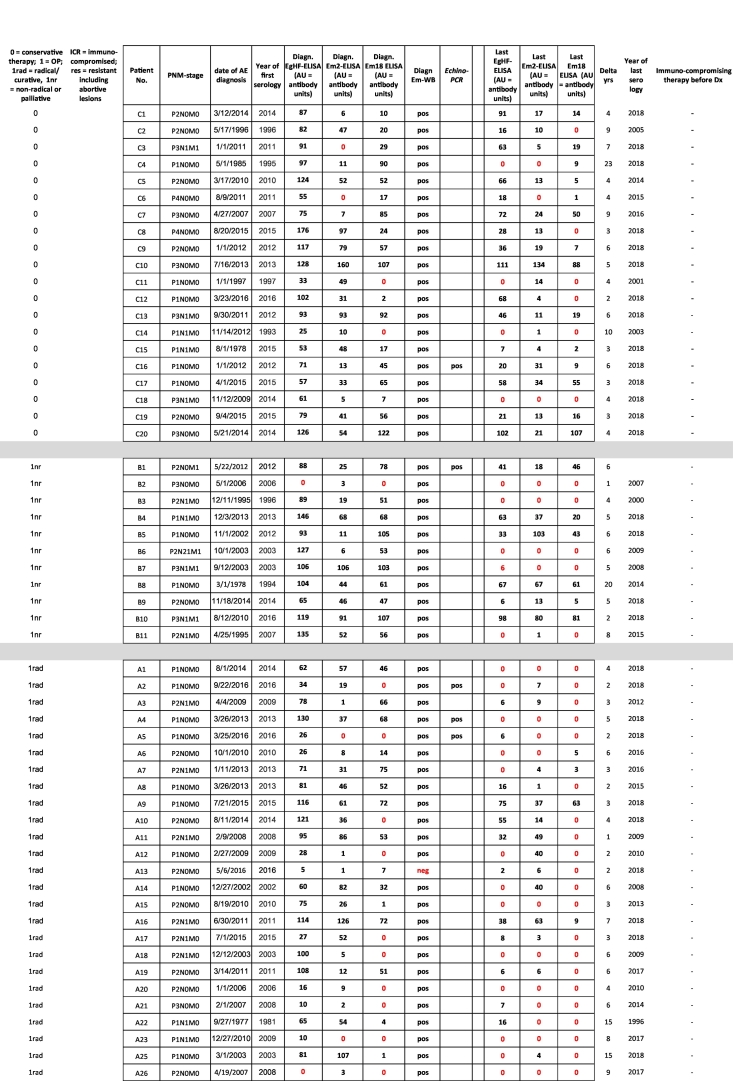

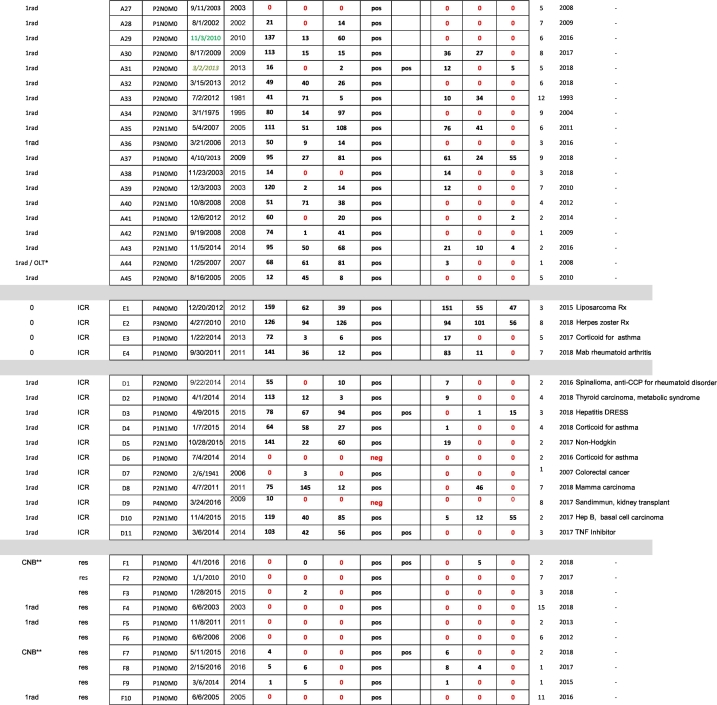
Baseline clinical characteristics of AE patients included in the study, plus all serological data. ELISA values: 0 = negative (red labelled); ≥1 = positive.

^⁎^Orthotropic Liver Transplantation. ^⁎⁎^Core needle biopsy.

**Fig. 1:**
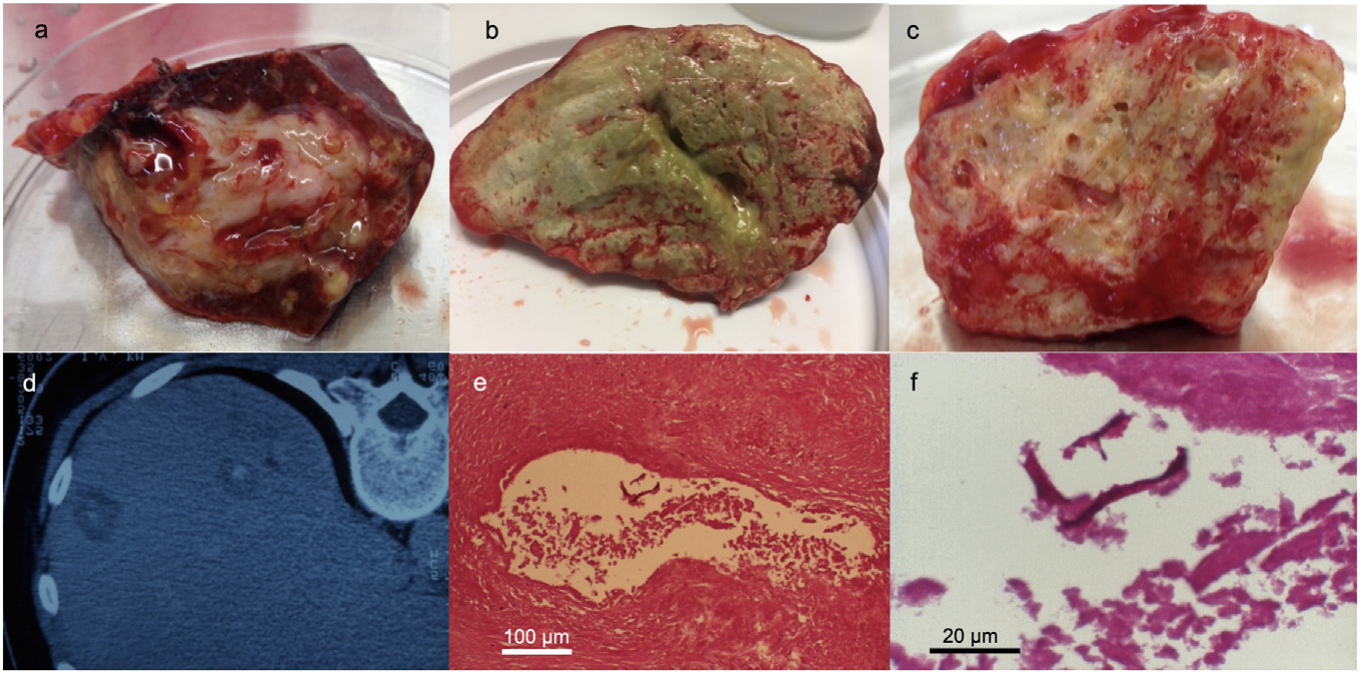
Exemplary macroscopic visualization of different lesion presentations in AE patients: [a] (patient A9) and [b] (patient A37) both show lesions from AE patients that had a radical (curative) resection reported by the surgeons, but who maintained a recEm18-seropositivity until the end of the present study period. [c] (patient D3) shows part of the resected material that was, according to the surgeons, radically resected from an immunocompromised AE patient, but who nevertheless maintained a recEm18-seropositivity until the end of the study period. [d] (patient F2) shows CT-findings of two small lesions that were considered as putatively “abortive”; the patient remained stable for a period of 7 years (= until the end of the study period). [e & f] (patient F4) shows histological analyses of the resected “abortive” material, HE-staining for [e], and PAS-staining for [f], where fragments of the PAS-positive laminated layer can still be seen.

Selected exemplary macroscopic and microscopic presentations of different lesions in AE patients are shown in Fig. 1.

### 2.2 Materials and methods

#### 2.2.1 EgHF-, Em2- and recEm18-ELISA

The ELISAs were carried out essentially as described by Gottstein et al. (1993), by testing sera at a 1: 100 dilution and using the following antigens (at optimized coating concentrations): EgHF-antigen (5 μg protein per ml) (antigen source/production as referenced in Müller et al., 2007); Em2 antigen (2.8 mg carbohydrate per ml) (antigen source/production as referenced in Gottstein et al., 1983); recEM18-antigen (1 μg protein per ml) (antigen source/production as referenced in Ito et al., 2002). All three *Echinococcus*-ELISAs were run under daily routine conditions (accredited according to ISO 17025 norms) and were quantitatively calibrated by employing a previous serum from a given patient for testing a subsequent one at a later time point. Inter-test and intra-test variations were assessed by determining the coefficients of variation for reference negative and positive sera, all having been tested in triplicates on each test plate.

#### 2.2.2 Em-Immunoblottting

Immunoblotting was performed as described previously for *E*. *granulosus* hydatid fluid antigen (Poretti et al., 1999), with the exception that EmVF (7 μg per cm slot) was used instead of EgHF, such as described by Müller et al. (2007). Sera were tested at a dilution of 1:10. Interpretation criteria were as previously described (Müller et al., 2007). Seropositivity of the immunoblotting relied primarily on signals detected against the immunoreactive band triplet of 20–22 kDa and the specific 8 kDa band associated to an antigen B subunit.

### 2.3 Statistical analyses

The first (if applicable pre-surgical) time point serum available from each patient was used for the estimation of the diagnostic sensitivities and 95% confidence intervals (95% CI) of the three antigens used in ELISA and of the Em-Immunoblot (One Proportion method).

The follow-up analysis was conducted considering the first test available before surgical or chemotherapeutical treatment as baseline. It included all serum samples of a patient up to the last (most recent) serum sample available within the frame of this study. Among patients who were positive at baseline, we compared rates of negativization between cured and non-cured patients in those who underwent surgical treatment, and between patients with good (putatively curative) and poor response in those treated with ABZ. Here the comparison between 2 groups was done with the Two Independent Proportion method.

We compared the shortest time spans needed for recEm18-negativization by AE patients with a radical surgical treatment with the time spans found in those AE patients with non-radical, palliative surgery or ABZ treatment who were able to negativize during the study period. This was done using a Student's *t*-test (type 2).

*p*-Values <0.05 were considered statistically significant. All statistical analyses were performed using the NCSS 12.0.9 software.

## 3 Results

### 3.1 Diagnostic sensitivities

To evaluate the diagnostic sensitivities of the 4 serological tests in relation to clinically evident AE (groups A-E), the first available serum from each patient with AE was used (Table 2). For all 91 AE patients of groups A-E, the Em-Immunoblot showed the best overall diagnostic sensitivity (98%), followed by the EgHF-ELISA (95%), and then the Em2-ELISA (86%) and finally the recEm18-ELISA (80%) (Table 2). The lowest diagnostic sensitivities were found in patients undergoing radical surgery, i.e. groups A and D, with 84% and 64%, respectively, for the Em2-ELISA and 73% in both groups for the recEm18-ELISA (Table 2). Statistically, the diagnostic sensitivity of the Em-Immunoblot was significantly higher than those of the Em2-ELISA (*p* = 0.0089) and of the recEm18-ELISA (*p* = 0.0005), but not when compared to the EgHF-ELISA (*p* = 0.4696). Similarly, the diagnostic sensitivity of the EgHF-ELISA was significantly higher than those of the Em2-ELISA (*p* = 0.047) and of the recEm18-ELISA (*p* = 0.0037). Differences in diagnostic sensitivity between the Em2-ELISA and the recEm18-ELISA were not significant (*p* = 0.3242).

**Table 2:** Diagnostic sensitivities of the four serological tests investigated, referring individually to groups A to F, and overall sensitivities for all 91 AE patients including included in groups A to E (shown in bold/italic), or all 101 AE patients included in groups A to F (shown in bold/italic), respectively [CI95%].

group (n)	EgHF-ELISA (%)	Em2-ELISA (%)	recEm18-ELISA (%)	Em-Immunoblot (%)
A (n = 45)	96 [84–99]	84 [71–94]	73 [58–85]	98 [88–100]
B (n = 11)	91 [59–100]	100 [71–100]	91 [59–100]	100 [71–100]
C (n = 20)	100 [83–100]	90 [68–99]	90 [68–99]	100 [83–100]
D (n = 11)	82 [48–98]	64 [631–89]	73 [39–94]	82 [48–98]
E (n = 4)	4/4 [40–100]	4/4 [40–100]	4/4 [40–100]	4/4 [40–100]
***A***-***E*** (***n*** = ***91***)	***95***	***86***	***80***	***98***
F (n = 10)	30 (7–65)	30 [7–65]	0 [0−32]	100 [70–100]
***A***-***F*** (***n*** = ***101***)	***88***	***80***	***72***	***98***

For group F (individuals presenting “abortive” lesions), it became evident that only the Em-Immunoblot was capable of serologically identifying such patients (respective diagnostic sensitivity: 100%), while all other tests demonstrated either very low sensitivities (30% for EgHF- and Em2-ELISA) or completely failed (recEm18-ELISA) (Table 2). Statistically, the diagnostic sensitivity of the Em-Immunoblot was significantly higher than that of the EgHF-ELISA (*p* = 0.0157), the Em2-ELISA (*p* = 0.0002) and the recEm18-ELISA (*p* = 0.0000). The diagnostic sensitivities regarding all 101 AE patients (groups A-F) included in this study are shown in Table 2.

### 3.2 Follow-up serological tests

The number and percentages of initially positive and subsequently negative samples in ELISA over time after treatment among the different patient groups are shown in Table 3. For this analysis, we included only those AE patients that were initially positive in a given test, because only under such circumstances a respective negativization could be documented.

**Table 3:** Negativization rate of initially seropositive AE patients following treatment (last serum available after start of treatment) (CI95%).

AE-group (group size)	no. of initially EgHF-positives	no. of EgHF-negatives at the end	% EgHF negativization (CI95%)	no. of initially Em2-positives	no. of Em2-negatives at the end	% Em2 negativization (CI95%)	no. of initially recEm18-positives	no. of recEm18-negatives at the end	% recEm18 negativization (CI95%)
A (n = 45)	43	21	49% (33–64%)	38	18	47% (31–64%)	33	25	76% (58–89%)
B (n = 11)	10	3	30% (7–65%)	11	4	36% (11–69%)	10	4	40% (12–74%)
C (n = 20)	20	4	20% (6–44%)	18	2	11% (1–35%)	18	4	22% (6–48%)
D (n = 11)	9	3	33% (8–70%)	7	4	57% (18–90%)	8	6	75% (35–97%)
E (n = 4)	4	0	0/4 (0–60%)	4	1	1/4 (1–81%)	4	2	2/4 (7–93%)
A-E (n = 91)	86	31	**36%**	78	29	**37%**	73	41	**56%**

For all 3 antigens tested, the highest rate of negativizing AE patients was found in group A (radical surgery). This was most evidenced in the recEm18-ELISA, which had a statistically significantly higher rate of negativization than the EgHF- and the Em2-ELISA, respectively. A similar performance was achieved in group D (immunosuppressed with radical surgery), while groups B (palliative surgery) and C (benzimidazoles only) showed less negativizing AE patients. Overall, the recEm18-ELISA was clearly more sensitive with regard to negativization-rate (41/73) as compared to the EgHF-ELISA (31/86) and the Em2-ELISA (29/78). Statistically, the rate of AE patients negativizing in the recEm18-ELISA was significantly higher than in the EgHF-ELISA (*p* = 0.0111) and also in the Em2-ELISA (*p* = 0.0194), while EgHF- and Em2-ELISA did not significantly differ in that aspect.

Remarkably, all AE-patients from group F were already initially negative in the recEm18-ELISA, indirectly confirming the association between an “abortive” AE and a negative recEm18-serology.

**Fig. 2:**
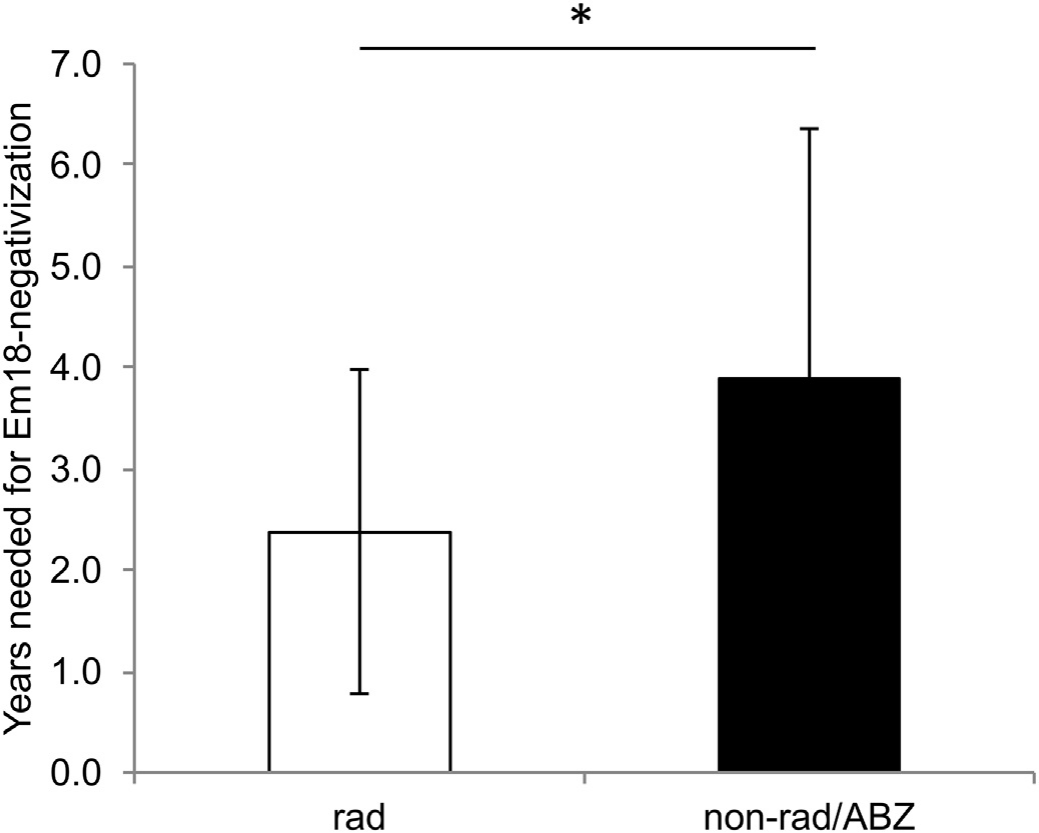
Comparison of the shortest time spans needed for recEm18-negativization by 26 AE patients with a radical surgical treatment (rad) with the time spans found in those 9 AE patients with non-radical, palliative surgery or ABZ treatment who were able to negativize during the study period (non-rad/ABZ). The difference was statistically significant (*p* = 0.0487).

Finally, the mean time for recEm18-negativization in AE patients who underwent radical surgery and for whom the respective data was available (i.e. 26 patients out of groups A and D) was 2.4 years (SD 1.6), while it took a mean of 3.9 years (SD 2.5) for those AE patients with non-radical, palliative surgery or ABZ treatment who were able to negativize during the study period (i.e. 9 patients from groups B, C and E) (Fig. 2). The difference was statistically significant (*p* = 0.0487).

## 4 Discussion

Substantial efforts have been undertaken to optimize the performance of serological tests for both, initial diagnosis of AE, as well as for long-time monitoring of peri- and/or post-therapeutical courses in AE patients (reviewed in Wen et al., 2019). The latter is of special interest in view of gaining predictive information about a successful, i.e. curative, outcome of treatment, which might contribute to an abrogation of the daily medication necessary to control AE in non-radically operated AE patients. There is also a considerable interest in having serological tests that can discriminate between active and inactive (“abortive” AE) lesions at diagnosis. This holds true especially when accidentally detecting non-symptomatic hepatic lesions upon routine imaging investigation of the liver. Such lesions may be suggestive for AE in the differential diagnosis, but the putative viability status can often not be determined by imaging procedures.

In the present study, we retrospectively evaluated serological data obtained by our routine diagnosis with sera from AE patients before and after curative or palliative surgical treatment and/or benzimidazole treatment, respectively. Due to the relatively stringent inclusion criteria for AE patients, some group sizes turned out rather small (groups D, E, F), thus limiting the informative power for these three groups. In our daily AE serology, we apply a combination of ELISAs based on antigens EgHF, Em2, and recEm18 as well as the highly sensitive Em-Immunoblotting based on EmVF. Referring to current literature (reviewed in Siles-Lucas et al., 2017), the combination of these tests offers a very high sensitivity and specificity for the diagnosis of human AE.

Our study offers the advantage of presenting laboratory data from patients who have been clinically carefully diagnosed and followed. By that, they represent the entire severity spectrum of the disease ranging from abortive to inoperable AE cases, the latter conventionally requiring life-long treatment with benzimidazoles (see Table 1). Furthermore, we present a similar range of AE manifestations in the growing group of immunocompromised patients that may represent specific challenges with regards to treatment and diagnosis. Thus, the dataset resulting from this study is perfectly suited to consolidate previous findings regarding the diagnostic operating characteristics of the abovementioned tests (reviewed in Siles-Lucas et al., 2017). In particular, our results suggest that Em-Immunoblotting represents the most sensitive test to diagnose active as well as inactive (“abortive”) AE cases, especially when linked to the ELISAs listed above. Overall, only three out of 101 AE patients were initially Em-Immunoblot negative. All three sera were also negative when tested in parallel by the presently only commercially available immunoblot tests for diagnostic AE serology, the Echinococcus-LD-BIO™ and the Echinococcus-Euroline™-Westernblot. However, remarkably, one of these three AE-patients yielded a unique positive recEm95 reaction in the Euroline™-Westernblot. Two of these completely seronegative AE patients belonged to the group of immunocompromised and radically-surgically-treated patients (D6 and D9).

Some patients of group A (radical surgery) failed to negativize in the recEm18-ELISA. Interestingly, for two of those patients we were able to macroscopically document the respective resected lesions and to reveal their challenging nature. The lesion shown in Fig. 1a (patient A9) included multiple small satellite lesions scattered within the periparasitic liver tissue. Such small lesions affecting other liver lobes may not be detected by surgeons prior to or during surgery, and therefore may not be removed. This may explain the observed persistence of anti-recEm18 antibodies in this patient. However, the lesions may be controlled by the post-operative ABZ administration and thus remain asymptomatic. Similarly, the lesion shown in Fig. 1b (patient A37) appears as a far advanced AE, where radical surgery may be hard to achieve. These two cases illustrate the continuous discussion about attempted radical surgery and potentially remaining lesions that could not be detected prior to and during surgery. Conclusively, a claimed radical resection that does not yield subsequent recEm18-negativity should be cautiously interpreted in view of a potential abrogation of ABZ medication. Summarized, as compared to both the EgHF- and Em2-ELISA, the recEm18-ELISA performed significantly better in follow-up AE serology and up to now can be considered the best laboratory tool to monitor a regressive and putatively curative course of AE in treated immunocompetent and immunocompromised patients. This finding matched those of others who already documented that seropositivity in the recEm18-ELISA reflects the presence of viable parasitic lesions (Ammann et al., 2004; Ishikawa et al., 2009; Tappe et al., 2010; Tappe et al., 2009; Bresson-Hadni et al., 2011). Nevertheless, recEm18-follow-up performance requires an initial recEm18-positivity, which was found in only 80% of the patients in our study. Therefore, the remaining 20% of AE patients could not be monitored with the recEm18 antigen. Alternative assays that may detect these recEm18-negative AE patients and reflect the course of disease are needed. Several other serological tests hold promise as tools to assess the efficacy of treatment (Siles-Lucas and Gottstein, 2001; Ito and Craig, 2003). For example as assessed by ELISA, a reduction of *Echinococcus*-specific serum IgE and IgG4 levels against crude *E*. *multilocularis* vesicle antigen were shown to be associated with absence of recurrence upon surgical and chemotherapeutical treatment of AE patients (reviewed in Gottstein et al., 2014). At the same time, re-emergence of specific IgG4 antibodies turned out to be indicative for recurrence of the infection. At least to a certain extent, these observations were confirmed by immunoblotting in that serum IgG4 from AE patients with progressive disease exhibited a distinct reactivity pattern with *E*. *multilocularis* antigen (reviewed in Siles-Lucas and Gottstein, 2001). Furthermore, progressive reduction of *E*. *multilocularis* metacestode-specific IgG1, IgG3, and IgE responses were found to be associated with regression of the disease from an active to a stable and cured stage (Huang et al., 2014). Conversely, reactivity of IgG2 and IgG4 against crude *E*. *multilocularis* vesicle antigen remained on comparatively high level in stable and progressive AE cases, whereas respective reactivity dropped in the cured form of the disease. It is evident that the novel analytical concepts listed above have to be further evaluated in terms of their suitability in follow-up examinations of post-treatment AE patients.

Currently, only the recEm18-ELISA-based follow-up AE serology has gained broad acceptance particularly in those labs that are specialized in diagnosis of human alveolar echinococcosis (reviewed in Siles-Lucas et al., 2017). It can be anticipated that new sets of native or recombinant antigens of *E*. *multilocularis* will be identified in the future that can complement recEm18 as biomarker to improve the serological assessment of AE patients. Nevertheless, this optimistic perspective does not alter the fact that serological follow-ups of AE must always be accompanied by periodic imaging of the lesions to achieve a reliable post-treatment monitoring of the disease. In this context we may emphasize on the fact that the test procedures investigated in this study are all in-house tests that are based on a very long-term development and were subsequently subjected to an ISO-17025-normed evaluation and validation process. Other diagnostic laboratories that are involved in AE serology may prefer to use similar but commercialized tests. With regard to commercialized ELISAs, the antigens we were using have been included in the so-called Em2plus-ELISA (Bordier Affinity Products, Crissier, Switzerland), which works on the basis of a combination of the Em2-antigen plus the recEm18-antigen. For follow-up purposes, the same company offers the Em18-ELISA, which works on the basis of the same recEm18-antigen we have been using. With regards to immunoblotting, two companies offer respective assays: the Echinococcus-LD-BIO™, which uses a crude extract of *E*. *multilocularis* metacestode as antigen, and the Echinococcus-Euroline™-Westernblot, which uses an in vitro produced *E*. *multilocularis* vesicular fluid antigen. According to our restricted experience, both tests perform excellently in the routine serodiagnosis of AE. Consequently, a diagnostic laboratory can base its methodology on very good and reliable commercial products.

Finally, the serological findings with regard to “abortive” cases of AE were very interesting, although we have to consider that this group was rather small, and that a definitive diagnosis of “abortive AE” could only be confirmed histologically in four of the 10 cases. However, all patients were Em-Immunoblot-positive and recEm18-negative, which perfectly fits with the non-viability situation. Only three out of these 10 patients showed a very weak anti-Em2-seropositivity. This anti-Em2-response could be explained by the continuous presence of a certain amount of remaining debris of the laminated layer (the major source of Em2-antigen) causing a continuous stimulation of the anti-Em2 response in some of the patients. Most likely there is a minimal amount of such laminated layer needed to cause and maintain an antibody response. A visualization of this hypothesis is presented in Fig. 1e & f (patient F4), where some laminated layer fragments (PAS-positive) can be seen. Nevertheless, this specific patient was diagnostically Em2-negative.

As a first conclusion, when assuming that exposure to *E*. *multilocularis* infection may frequently result in an abortive situation (Gottstein et al., 2015a; Gottstein et al., 2015b), the accidental finding of small intrahepatic lesions, especially when appearing calcified, by imaging procedures, may require an appropriate AE serology to help explain the etiology of these lesions, and to help appease the patient with a favorable diagnosis of “abortive” AE. Thus, unnecessary surgical or chemotherapeutical intervention can be avoided. Nevertheless, we will need more and longer experience with such cases to elaborate a protocol that allows the clinician to perform an early classification of such a case.

As a final conclusion, our data strongly support that all laboratories, also non-specialized ones, adopt a similar methodical strategy for AE serodiagnosis as shown here, i.e. to perform e.g. commercial ELISA-based *Echinococcus* spp. screening tests, and then to use a second confirmatory *E*. *multilocularis*-based immunoblot test (e.g. LD-BIO™ and Euroline™-Westernblot), while using the commercial recEm18-ELISA for follow-up studies.
